# Nordic diet, Mediterranean diet, and the risk of chronic diseases: the EPIC-Potsdam study

**DOI:** 10.1186/s12916-018-1082-y

**Published:** 2018-06-27

**Authors:** Cecilia Galbete, Janine Kröger, Franziska Jannasch, Khalid Iqbal, Lukas Schwingshackl, Carolina Schwedhelm, Cornelia Weikert, Heiner Boeing, Matthias B. Schulze

**Affiliations:** 1 Department of Molecular Epidemiology, German Institute of Human Nutrition Potsdam-Rehbruecke (DIfE), Nuthetal, Germany; 2 Department of Epidemiology, German Institute of Human Nutrition Potsdam-Rehbruecke, Nuthetal, Germany; 3 Department of Food Safety, Federal Institute for Risk Assessment, Berlin, Germany; 4 University of Potsdam, Institute of Nutritional Sciences, Nuthetal, Germany; 5 German Center for Diabetes Research (DZD), München-Neuherberg, Germany; 6 DZHK (German Centre for Cardiovascular Research), partner site Berlin, Berlin, Germany; 7 NutriAct – Competence Cluster Nutrition Research Berlin-Potsdam, Nuthetal, Germany

**Keywords:** Mediterranean diet, Nordic diet, regional diets, chronic diseases, diabetes, myocardial infarction, stroke, cancer, EPIC-Potsdam study, longitudinal analysis

## Abstract

**Background:**

The Mediterranean Diet (MedDiet) has been acknowledged as a healthy diet. However, its relation with risk of major chronic diseases in non-Mediterranean countries is inconclusive. The Nordic diet is proposed as an alternative across Northern Europe, although its associations with the risk of chronic diseases remain controversial. We aimed to investigate the association between the Nordic diet and the MedDiet with the risk of chronic disease (type 2 diabetes (T2D), myocardial infarction (MI), stroke, and cancer) in the EPIC-Potsdam cohort.

**Methods:**

The EPIC-Potsdam cohort recruited 27,548 participants between 1994 and 1998. After exclusion of prevalent cases, we evaluated baseline adherence to a score reflecting the Nordic diet and two MedDiet scores (tMDS, reflecting the traditional MedDiet score, and the MedPyr score, reflecting the MedDiet Pyramid). Cox regression models were applied to examine the association between the diet scores and the incidence of major chronic diseases.

**Results:**

During a follow-up of 10.6 years, 1376 cases of T2D, 312 of MI, 321 of stroke, and 1618 of cancer were identified. The Nordic diet showed a statistically non-significant inverse association with incidence of MI in the overall population and of stroke in men. Adherence to the MedDiet was associated with lower incidence of T2D (HR per 1 SD 0.93, 95% CI 0.88–0.98 for the tMDS score and 0.92, 0.87–0.97 for the MedPyr score). In women, the MedPyr score was also inversely associated with MI. No association was observed for any of the scores with cancer.

**Conclusions:**

In the EPIC-Potsdam cohort, the Nordic diet showed a possible beneficial effect on MI in the overall population and for stroke in men, while both scores reflecting the MedDiet conferred lower risk of T2D in the overall population and of MI in women.

**Electronic supplementary material:**

The online version of this article (10.1186/s12916-018-1082-y) contains supplementary material, which is available to authorized users.

## Background

In the last years, the Nordic diet has emerged as a healthy regional eating option [1]. The Nordic diet tries to reflect the diet consumed in Nordic countries, particularly its healthier choices, including the intake of apples, pears and berries, root and cruciferous vegetables as well as cabbages, whole grain and rye bread as cereals, high intake of fish, low-fat dairy products, potatoes and vegetable fats, among others [1, 2]. To our knowledge, the long-term effects of the Nordic diet on major chronic diseases have only been investigated in prospective cohorts within Nordic countries (Denmark, Sweden, and Finland) [3–10] and the observed effects remain inconsistent.

Another regional diet, the Mediterranean diet (MedDiet), has been extensively evaluated in relation to chronic diseases. The MedDiet refers to the dietary habits in countries around the Mediterranean basin, based on the rural models. This diet postulates a high intake of fruits, nuts and seeds, vegetables, fish, legumes, cereals, low intake of meat and dairy products, moderate intake of alcohol, mainly from red wine, and extra virgin olive oil as a major fat source [11]. A large multicenter trial conducted in Spain, the PREDIMED (*Prevención con dieta mediterránea*) study, proved beneficial health effects of the MedDiet, primarily in the prevention of cardiovascular disease (CVD) [12], and similar effects have also been observed in prospective studies [13, 14]. The PREDIMED study also showed a reduction of the risk of type 2 diabetes (T2D) [15] and a reduction of incident breast cancer [16]. For T2D, this association was also confirmed in large prospective studies and meta-analyses [17–19]. Further, some beneficial effects have been also shown for certain types of cancer [20].

However, there is some controversy on the possible beneficial effects of the MedDiet outside of the Mediterranean region [21]. As stated by Hoffman and Gerber [21], certain factors, such as food availability, composition of foods available in the Mediterranean and non-Mediterranean countries, and culinary traditions, could complicate following a MedDiet. Similarly, the same could be stated for the application of the Nordic diet in a non-Nordic area. The EPIC-Potsdam (European Prospective Investigation into Cancer and Nutrition-Potsdam) cohort presents a suitable frame for the evaluation of the possible beneficial effects of these two regional diets on the onset of major chronic diseases in both non-Mediterranean and non-Nordic populations. Thus, we aimed to evaluate the association between the Nordic diet and the MedDiet with the risk of major chronic diseases (T2D, myocardial infarction (MI), stroke, and cancer) in the EPIC-Potsdam population.

## Methods

### Study population and design

The EPIC-Potsdam study is a prospective cohort based in Germany as part of the European-wide multicenter EPIC study. Briefly, it includes 27,548 men and women, aged mainly 35–65 years, recruited between 1994 and 1998. Participants were selected from the general population of Potsdam and surroundings and were invited to a baseline examination conducted by trained staff. At baseline, computer-guided interviews on lifestyle and medical history were also conducted. Every 2–3 years, participants received via mail a follow-up questionnaire in order to assess incident disease cases. The study was approved by the Ethical Committee of the Federal State of Brandenburg and written informed consent was obtained from all participants at the time of recruitment [22, 23].

Those participants who reported prevalent T2D, MI, stroke, or cancer were excluded, as well as those with missing follow-up time, those with missing information on diet, and those who reported implausibly high or low energy intake (< 800 or > 6000 kcal/day). Finally, the analytical dataset included 9128 men and 14,357 women (*n* = 23,485). For the analysis on incident T2D, cases of other types of diabetes were excluded as well as those without verified T2D (final sample *n* = 23,411). In the case of the analysis for MI, stroke, and cancer, participants with non-verified positive self-reports were excluded, resulting in analytical samples of 23,409 for MI, 23,277 for stroke, and 23,152 for cancer.

### Dietary assessment and diet scores

Habitual dietary intake was assessed at baseline by self-administered semiquantitative food-frequency questionnaires (FFQ). This consisted of 148 food items and inquired after the frequency of intake and portion sizes during the last 12 months. Questions about the fat content of the dairy products consumed as well as about the types of fats used for preparation and cooking were included. The reproducibility and validity of the FFQ have been previously reported [24, 25]. Briefly, a total of 104 men and women, aged 35–64 years, completed 12 monthly 24-h dietary recalls, and these were used as a reference method for the estimation of the relative validity of the FFQ. Spearman correlations between FFQ and the mean intake of the 24-h dietary recalls were calculated, ranging from 0.14 for legumes to 0.90 for alcoholic beverages. For bread, the correlation was 0.51, 0.42 for cereals, 0.50 for fruits, 0.34 for vegetables, 0.37 for potatoes, 0.18 for nuts and seeds, 0.56 for milk and milk products, 0.21 for fish, 0.53 for meat, and 0.57 for processed meat. Reproducibility was calculated by administering the FFQ at 6-month intervals in the same study sample. In this case, correlations of food group intake ranged from 0.49 for bread to 0.89 for alcoholic beverages (median = 0.70). For cereals, the calculated correlation was 0.73, 0.61 for fruits, 0.54 for vegetables, 0.70 for legumes, 0.71 for potatoes, 0.66 for nuts and seeds, 0.55 for milk and milk products, 0.77 for fish and for meat, and 0.73 for processed meat [24].

The construction of the Nordic diet score was based on previous publications [1, 5, 26–29], as well as on the data available in the FFQ. We created a score ranging from 0 to 18 points, incorporating 9 components (Table 1). After categorizing each food component into sex-specific tertiles of intake, the participants received a score of 0 to 2 points according to the first, second, and third tertile, respectively. Nine components were included, namely whole grain and rye bread, berries, apples and pears, fish, cabbage and cruciferous vegetables, root vegetables, low-fat dairy products, potatoes, and vegetable fats (excluding olive oil).

**Table 1:** Components and food items considered the score created to evaluate adherence to the Nordic diet

Food groups	Food items considered in each food group	Scoring criteria
Whole grain and rye bread	Whole grain bread, ‘brown’, rye, mix bread and grain flakes, grains, muesli	Sex-specific tertiles T1 = 0, T2 = 1, T3 = 2
Berries	Currants, blackberries, raspberries	Sex-specific tertiles T1 = 0, T2 = 1, T3 = 2
Apple and pear	Apple, pear	Sex-specific tertiles T1 = 0, T2 = 1, T3 = 2
Fish	Fish (preserved and smoked is also considered)	Sex-specific tertiles T1 = 0, T2 = 1, T3 = 2
Cabbage and cruciferous vegetables	Broccoli, red cabbage, cauliflower, white cabbage, sauerkraut, radish, coleslaw (*0.5)	Sex-specific tertiles T1 = 0, T2 = 1, T3 = 2
Root vegetables	Carrots, celery, salsify, radish, coleslaw (*0.5)	Sex-specific tertiles T1 = 0, T2 = 1, T3 = 2
Dairy products	Low-fat dairy products, high fat dairy products, low-fat cheese, high-fat cheese	Sex-specific tertiles T1 = 0, T2 = 1, T3 = 2
Potatoes	All kind of potatoes	Sex-specific tertiles T1 = 0, T2 = 1, T3 = 2
Fats	Overall vegetable fats (margarine + vegetable oil, olive oil not included)	Sex-specific tertiles T1 = 0, T2 = 1, T3 = 2

Two different scores for the MedDiet were calculated. The traditional MedDiet score (tMDS) was originally proposed by Trichopoulou et al. [30] and slightly modified for non-Mediterranean populations [21]. Our study used a linear scale that incorporated nine key components, with a maximum of 18 points (Table 2). Each component was categorized into sex-specific tertiles of intake, except for alcohol and olive oil. For the five components presumed to reflect healthy components of the MedDiet (fruit, vegetables, legumes, fish, and cereals), 0 points were assigned to those participants in the lowest tertile of intake, 1 for those in the middle tertile, and 2 points to those in the highest tertile. This scoring was inverted for meat and dairy products. Alcohol intake was considered optimal if consumed in moderation (2 points for consumption of 5–25 g/d for women and 10–50 g/d for men, 0 otherwise). In the case of olive oil, the scoring was adapted for the intake in non-Mediterranean countries as follows: 0 points were assigned to non-consumers and sex-specific medians were calculated within olive oil consumers. Two points were assigned to those with consumption over the median and 1 point to those below [17, 31].

**Table 2:** Components and food items considered in the score created to evaluate adherence to the Mediterranean diet score (tMDS) based on that established by Trichopoulou et al. [30]

Food groups	Food items considered in each food group	Scoring criteria
Cereals	Whole grain bread, other bread, grain flakes, grains, muesli, cornflakes, crisps, pasta, rice	Sex-specific tertiles T1 = 0, T2 = 1, T3 = 2
Fruits and nuts	Fresh fruit, nuts	Sex-specific tertiles T1 = 0, T2 = 1, T3 = 2
Vegetables	Raw vegetables, green salad, cruciferous vegetables, cooked vegetables, garlic, mushrooms	Sex-specific tertiles T1 = 0, T2 = 1, T3 = 2
Fish	Fish (preserved and smoked is also considered)	Sex-specific tertiles T1 = 0, T2 = 1, T3 = 2
Legumes	Legumes (green peas, green beans, lentil, peas, bean stew)	Sex-specific tertiles T1 = 0, T2 = 1, T3 = 2
Meat	Poultry, meat, meat products	Sex-specific tertiles T1 = 2, T2 = 1, T3 = 0
Dairy products	Butter, low-fat dairy products, high fat dairy products, low-fat cheese, high-fat cheese	Sex-specific tertiles T1 = 2, T2 = 1, T3 = 0
Alcohol	Beer, wine, spirits, other alcoholic beverages	5 to 25 g/day for women = 2 10–50 g/day for men = 2 Outside of the range = 0
Olive oil	Olive oil for salad dressing, preparation of vegetables, and preparation of meat	Non-consumers = 0 < sex-specific median = 1 ≥ sex-specific median = 2

The second score applied to assess the adherence to the MedDiet was based on the Mediterranean pyramid (MedPyr) recommendations [11], and calculated following the algorithm developed by Tong et al. [14]. This score categorizes 15 foods or food groups into those that should be consumed in high quantities, in moderation, or in low quantities. The pyramid also provides information on the number of servings recommended, per week or per day. Vegetables, legumes, and fish are food groups advised to be consumed in high quantities. Fruits, nuts, cereals, dairy products, white meat, egg, and alcohol are considered foods that should be consumed in moderation. Red meat, processed meat, potatoes, and sweets are recommended to be consumed in low quantities. Olive oil should represent the main fat source. Detailed information on the calculation of this score is provided in Table 3.

**Table 3:** Scoring criteria for the construction of the MedPyramid score (from Tong et al. [14])

Component	Recommended intake^a^	Score of 0^a^	Score of 1^a^
Vegetables^b^	≥ 6 /d	0 /d	≥ 6 /d
Legumes^b^	≥ 2 /wk	0 /wk	≥ 2 /wk
Fruits^c^	3–6 /d	0 /d	3–6 /d
Nuts^c^	1–2 /d	0 /d	1–2 /d
Cereals^c^	3–6 /d	0 /d	3–6 /d
Dairy^c^	2 /d	0 /d	1.5–2.5 /d
Fish^b^	≥ 2 /wk	0 /wk	≥ 2 /wk
Red meat^e^	˂ 2 /wk	≥ 4 /wk	˂ 2 /wk
Processed meat^e^	≤ 1 /wk	≥ 2 /wk	≤ 1 /wk
White meat^c^	2 /wk	0 /wk	1.5–2.5 /wk
Egg^c^	2–4 /wk	0 /wk	2–4 /wk
Potatoes^e^	≤ 3 /wk	≥ 6 /wk	≤ 3 /wk
Sweets^e^	≤ 2 /wk	≥ 4 /wk	≤ 2 /wk
Alcohol^d^	10–50 g/d for men, 5–25 g/d for women	> 50 g/d for men, > 25 g/d for women	10–50 g/d for men, 5–25 g/d for women
Olive oil^f^	Principal source of dietary lipids	Non-consumers	Consumers

^a^All recommendations are in number of servings per day or per week and we used continuous scoring for all components, except for olive oil and alcohol

^b^For those components for which a high consumption was recommended, continuous scores from 0 to 1 were assigned proportionally from no consumption to meeting the recommended level of consumption

^c^For components for which moderate consumption was recommended, we assigned a score of 1 for consumption within the recommended levels and 0 for no consumption, with consumption levels in between scored proportionately; overconsumption (double the mid-point value of the recommended intake) was penalized and received a maximum score of 0.5, with consumption between the recommended level and the penalty point scored proportionally

^d^For alcohol, we assigned a score of 1 for consumption levels within recommendation, non-consumption was scored 0.5 while overconsumption was scored 0

^e^For those components for which a low consumption was recommended, consumption below the recommended levels was assigned a score of 1 and double the recommended levels were assigned a score of 0; levels in between scored proportionally

^f^For olive oil, all non-consumers were scored 0 and all consumers 1

### Outcome ascertainment

Information on incident chronic diseases (T2D, MI, stroke, and cancer) was obtained from self-reports during follow-up. These included self-reports on the respective condition, relevant medication, or reason for a reported change in diet. Further diagnoses were found by record linkages with the Common Cancer registry of the different Federal States [32]. Moreover, all potential incident cases were verified by contacting physicians, local cancer registries, and information of death certificates. Incident cases were coded based on the International Classification of Diseases (ICD-10 codes: I21 for myocardial infarction, I60, I61, I63, I64 for stroke, E11 for T2D, and C00–97 for cancer (except C44: non-melanoma skin cancer)). We considered data until the end of the fifth follow-up period (year 2009). Fatal cases due to MI or stroke were also included as incident cases.

### Covariate assessment

General characteristics on sociodemographic, lifestyle, and health status were assessed at baseline using self-administered questionnaires. Educational attainment was categorized as (1) currently in training/no certificate or skill, (2) professional school (vocational training), and (3) college or higher education. Smoking status of the participants was categorized as never smoker, former smoker, current smoker, and smoking ≥ 20 cigarettes/day. Physical activity was defined as the mean time spent on leisure time physical activities and cycling (hours/week) during summer and winter, and further categorized (cycling: 0, > 0–2.5, > 2.5–5; > 5 h/week, and sports: 0, > 0–4; > 4 h/week). At baseline examination weight, height and waist circumference were measured by trained staff. Prevalent hypertension diagnosis was based on blood pressure measured at baseline and self-report medication. Total energy intake (kcal/day) was calculated from the FFQ.

### Statistical analysis

Cox proportional hazard regression models were used to investigate the association of the three different dietary pattern scores and the risk of incident chronic diseases (T2D, MI, stroke, and cancer, separately). Multivariable-adjusted hazard ratios (HRs) and 95% confidence intervals (CIs) were calculated across tertiles of dietary pattern scores, as well as considering the patterns as continuous variables (by 1 standard deviation (SD) and per 1 unit increment of the scores). The dependent time variable was defined as the time period between the age of recruitment and the age of exit (age of diagnoses or date of death or censoring). In order to be less sensitive to violations of the HR, the models were stratified by age in years. Two models were applied; the first adjusted for sex and the second additionally adjusted for body mass index (kg/m^2^), waist circumference (cm) using the residual method adjusted for body mass index, total energy intake (kcal/day), education, smoking status, cycling, sports, and vitamin supplementation (yes/no). In addition, alcohol intake was considered in the analysis of the Nordic diet (0, > 0–6, > 6–12, > 12–24, > 24–60, > 60–96 g/d; plus one more category only for men, > 96 g/d). Prevalent hypertension status (yes/no) was also included in the analysis on T2D, MI, and stroke. Non-linear associations between the a priori defined scores and outcomes of interest were investigated using restricted cubic splines.

To determine the relative impact of the different components comprising the three dietary patterns on the observed associations, dietary scores were calculated alternately omitting each single component and adjusting Cox models for the excluded component.

Potential effect modification by sex was assessed by evaluating the cross-product term between sex and dietary pattern scores in the fully adjusted model. In case interactions were detected, stratified analyses by sex were applied.

Regression-based multiple imputation was conducted to impute missing information on covariates. Five imputed datasets were created. For the main analysis on the association of the patterns with the risk of onset of chronic diseases, missing covariates were observed in 2.5% of participants. Sensitivity analyses were performed by excluding participants with incident cases in the first 2 years of follow-up in the longitudinal analyses. In the analysis of the association of the dietary patterns with stroke, additional analyses were performed excluding incident hemorrhagic stroke cases.

## Results

### Cohort and dietary pattern characteristics

Sociodemographic lifestyle and anthropometric characteristics according to adherence to the three different diet scores are presented in Table 4. Participants with higher adherence to the Nordic diet where older than those with a low adherence, presented higher waist circumference, were more likely to be overweight and hypertensive, and less likely to be current or former smokers. They were also physically more active. Participants with higher scores for the tMDS diet were older, more likely to be men, higher educated, and physically more active than those with lower adherence. For the MedPyr score, participants with higher adherence were younger, less likely to be hypertensive, more likely with a higher education, and physically more active that those in the lowest tertile of adherence. All three diet scores were associated with each other, observing a stronger association between the two MedDiet scores and a lower one between the Nordic diet and the MedPyr scores (Additional file 1: Table S1). Apart from the differences directly related to the calculation of the scores, further differences were observed. Total energy and fiber intake were positively associated with the three scores, and somewhat more pronounced for the Nordic diet. Concerning macronutrient intake, higher intake of protein from plants was observed in participants with higher adherence to the Nordic diet than in those with a lower adherence; this was also observed for polyunsaturated fat intake. Participants with a higher score for the tMDS had lower intake of saturated fat. Concerning food intake, higher adherence to the Nordic diet was also associated with higher intake of legumes and higher intake of sweets. Higher scores for the tMDS and the MedPyr were also positively associated with sweets, whole grain cereals, and alcohol intake. Intake of potatoes was positively associated with the tMDS but inversely with the MedPyr score.

**Table 4:** General characteristics of the EPIC-Potsdam population according to the degree of adherence to diet scores

		Nordic diet score		tMDS score		MedPyr score	
Variable	Total population (*n* = 23,485)	Low adherence (*n* = 7686) 0–7 points	High adherence (*n* = 7637) 11–18 points	Low adherence (*n* = 7077) 0–7 points	High adherence (*n* = 6709) 11–18 points	Low adherence (*n* = 7828) < 6.2 points	High adherence (*n* = 7828) > 7.3 points
Sex (% of male)	38.9	39.0	39.3	35.8	41.1	38.8	38.1
Age at recruitment	49.8 (8.9)	48.5 (8.6)	50.8 (8.9)	49.1 (9.0)	50.1 (8.8)	50.7 (9.1)	48.9 (8.6)
BMI (kg/m^2^)	26.1 (4.2)	25.8 (4.2)	26.3 (4.2)	26.1 (4.4)	26.0 (4.1)	26.2 (4.4)	26.0 (4.1)
% BMI ≥ 25 kg/m^2^	55.9	53.1	58.1	55.4	54.8	56.6	55.0
Waist circumference (cm)	85.6 (12.8)	84.8 (12.9)	86.4 (12.5)	85.2 (13.0)	85.5 (12.7)	86.0 (12.9)	85.2 (12.8)
Prevalent hypertension (%)	46.1	43.4	48.9	44.6	46.9	47.5	44.9
Current or former smokers (%)	51.2	57.4	45.5	52.2	50.0	50.6	51.4
Education; university degree (%)	37.7	36.5	39.0	30.9	44.6	29.5	44.9
Total sport (h/week)^a^	2.0 (0.0–4.0)	1.5 (0.0–3.5)	2.0 (0.5–5.0)	1.5 (0.0–3.5)	2.0 (0.5–5.0)	1.5 (0.0–3.5)	2.0 (0.5–4.5)

Data are shown as mean (SD) unless otherwise stated

*BMI* body mass index, *MedPyr* Mediterranean diet score based on the Mediterranean Pyramid, *tMDS* Mediterranean diet score based on that established by Trichopoulou et al. [30]

^a^Data are shown as median (IQR)

### Dietary patterns and incidence of major chronic diseases

Table 5 shows the associations observed between the a priori defined diet scores and the four different outcomes considered (T2D, MI, stroke, and cancer). The cubic spline analysis did not show evidence for non-linearity for any of the diet score–chronic disease associations investigated. During 246,219 person-years of follow-up, 1376 participants reported a new diagnosis of T2D (mean follow-up of 10.5 years). Both MedDiet scores were associated with lower risk of T2D in the fully adjusted model. Participants in the highest tertile of adherence to the MedPyr presented 20% lower risk of T2D in comparison with those in the lowest tertile (HR_T3 vs. T1_ 0.80, 95% CI 0.70–0.92; HR_per 1 SD_ 0.92, 95% CI 0.87–0.97). For the tMDS, participants with higher adherence presented 16% reduction of the risk (HR_T3 vs. T1_ 0.84, 95% CI 0.73–0.97; HR_per 1SD_ 0.93, 95% CI 0.88–0.98). No association was observed for the Nordic diet on the risk of incident T2D. In the analysis for MI, during 249,262 person-years, 312 participants developed MI (10.8 years follow-up on average). For the Nordic diet, a statistically not-significant inverse association with the onset of MI was observed (HR_T3 vs. T1_ 0.88, 95% CI 0.64–1.20; HR_per 1 SD_ 0.91, 95% CI 0.80–1.04). In the case of the MedDiet scores, an inverse but not-statistically significant association was observed for the MedPyr in the model corrected for sex and age. However, after the full adjustment for covariates, the association was attenuated, yet still suggesting a lower risk for higher adherence (HR_T3 vs. T1_ 0.84, 95% CI 0.63–1.11; HR_per 1 SD_ 0.92, 95% CI 0.82–1.04). For stroke, during 252,457 person-years follow-up, 321 new cases were identified (mean follow-up of 10.8 years), but no associations were observed. For cancer, from 244,339 person-years of follow-up, 1618 participants developed cancer (mean follow-up of 10.6 years). Similar to stroke, no associations were observed for overall cancer risk. Further sensitivity analysis excluding incident cases which occurred in the first 2 years of follow-up provided similar associations (Additional file 1: Table S2). For stroke, the exclusion of the hemorrhagic stroke cases did not show different associations (Additional file 1: Table S3). Stratified analyses were performed for sex when interaction between sex and the dietary patterns were detected in the fully adjusted model (Table 6). This was observed in the case of the Nordic diet on its association with stroke (*p* = 0.071). The stratified analyses revealed a decreased risk only in men (HR_T3 vs. T1_ 0.85, 95% CI 0.56–1.29; HR_per 1 SD_ 0.88, 95% CI 0.74–1.04). Possible interaction was also detected between sex and the MedPyr score for the risk on MI (*p* = 0.070). After stratification, an inverse association was observed in women (HR _T3 vs. T1_ 0.77, 95% CI 0.45–1.30; HR_per 1 SD_ 0.81, 95% CI 0.65–1.00), but not in men. Sensitivity analyses omitting individual components in the calculation of the scores were performed to test the dependence of observed associations on these components (Table 7). These analyses revealed that, for T2D, the omission of alcohol in both MedDiet scores attenuated the associations from a HR_per 1 SD_ of 0.93 (95% CI 0.88–0.98) to 0.95 (95% CI 0.90–1.01) in the case of the tMDS, and from 0.92 (95% CI 0.87–0.97) to 0.94 (95% CI 0.89–0.99) for the MedPyr score. The omission of alcohol in the calculation of the MedPyr score also led to the strongest attenuation of the association between this score and MI among women, from a HR_per 1 SD_ of 0.81 (95% CI 0.65–1.00) to 0.88 (95% CI 0.71–1.08). Similar results were observed for the exclusion of potatoes and red meat. However, the opposite was observed for olive oil, where, when excluded, the inverse association with MI became stronger (HR_per 1 SD_ 0.77, 95% CI 0.62–0.94).

**Table 5:** Prospective associations between adherence to the diet scores and the incidence of major chronic diseases

	Low adherence	Moderate adherence		High adherence		*p* for trend	per 1 SD		per 1 unit	
	(Ref.)	HR	95% CI	HR	95% CI		HR	95% CI	HR	95% CI
DIABETES										
Nordic diet										
Cases, n/person-year	413/80,081	494/85,511		469/80,627						
Model 1	1.00	1.04	0.91–1.18	1.00	0.87–1.14	1.000	1.00	0.94–1.05	1.00	0.98–1.02
Model 2	1.00	1.02	0.89–1.17	1.01	0.87–1.18	0.827	1.00	0.94–1.07	1.00	0.98–1.02
tMDS										
Cases, n/person-year	445/73,939	578/101,702		353/70,578						
Model 1	1.00	0.87	0.77–0.99	0.75	0.65–0.87	< 0.001	0.89	0.84–0.94	0.96	0.94–0.98
Model 2	1.00	0.92	0.81–1.04	0.84	0.73–0.97	0.019	0.93	0.88–0.98	0.97	0.95–0.99
MedPyr										
Cases, n/person-year	549/81,156	448/82,176		379/82,887						
Model 1	1.00	0.84	0.74–0.96	0.75	0.65–0.84	< 0.001	0.88	0.84–0.93	0.90	0.87–0.94
Model 2	1.00	0.90	0.79–1.02	0.80	0.70–0.92	0.001	0.92	0.87–0.97	0.93	0.89–0.97
MYOCARDIAL INFARCTION										
Nordic diet										
Cases, n/person-year	94/81,983	123/87,572		95/82,706						
Model 1	1.00	1.11	0.85–1.45	0.85	0.64–1.14	0.272	0.91	0.81–1.02	0.97	0.94–1.01
Model 2	1.00	1.13	0.85–1.49	0.88	0.64–1.20	0.440	0.91	0.80–1.04	0.97	0.93–1.01
tMDS										
Cases, n/person-year	91/75,908	137/104,226		84/72,128						
Model 1	1.00	0.99	0.76–1.29	0.84	0.62–1.13	0.283	0.91	0.81–1.02	0.97	0.93–1.01
Model 2	1.00	1.06	0.81–1.39	0.95	0.70–1.28	0.816	0.95	0.85–1.07	0.98	0.94–1.03
MedPyr										
Cases, n/person-year	122/83,499	105/84,148		85/84,614						
Model 1	1.00	0.89	0.69–1.16	0.78	0.59–1.03	0.073	0.89	0.80–1.00	0.91	0.83–1.00
Model 2	1.00	0.91	0.70–1.19	0.84	0.63–1.11	0.224	0.92	0.82–1.04	0.94	0.85–1.03
STROKE										
Nordic diet										
Cases, n/person-year	108/81,956	100/87,741		113/82,760						
Model 1	1.00	0.78	0.59–1.02	0.87	0.66–1.13	0.297	0.92	0.82–1.03	0.97	0.94–1.01
Model 2	1.00	0.82	0.62–1.09	0.97	0.72–1.31	0.819	0.97	0.85–1.10	0.99	0.95–1.03
tMDS										
Cases, n/person-year	91/75,836	144/104,416		86/72,204						
Model 1	1.00	1.06	0.82–1.38	0.91	0.68–1.22	0.498	0.99	0.88–1.11	1.00	0.96–1.04
Model 2	1.00	1.10	0.85–1.44	0.98	0.72–1.32	0.832	1.02	0.91–1.14	1.01	0.96–1.05
MedPyr										
Cases, n/person-year	124/83,599	93/84,291		104/84,566						
Model 1	1.00	0.80	0.61–1.04	0.96	0.74–1.25	0.701	0.97	0.87–1.09	0.98	0.90–1.07
Model 2	1.00	0.83	0.63–1.09	1.03	0.79–1.35	0.870	1.01	0.90–1.13	1.01	0.92–1.10
CANCER										
Nordic diet										
Cases, n/person-year	503/79,442	559/85,025		556/79,872						
Model 1	1.00	0.93	0.83–1.05	0.94	0.83–1.06	0.243	0.97	0.92–1.01	0.99	0.97–1.00
Model 2	1.00	0.95	0.84–1.08	0.99	0.87–1.14	0.774	0.99	0.93–1.05	1.00	0.98–1.01
tMDS										
Cases, n/person-year	482/73,638	691/100,771		445/769,931						
Model 1	1.00	0.98	0.87–1.10	0.91	0.80–1.03	0.119	0.97	0.92–1.02	0.99	0.97–1.01
Model 2	1.00	1.00	0.89–1.12	0.95	0.83–1.08	0.345	0.99	0.94–1.04	0.99	0.98–1.01
MedPyr diet										
Cases, n/person-year	571/80,743	556/81,584		491/82,013						
Model 1	1.00	1.03	0.92–1.16	0.96	0.85–1.09	0.636	0.99	0.94–1.04	0.99	0.95–1.03
Model 2	1.00	1.04	0.93–1.17	1.00	0.88–1.13	0.979	1.01	0.96–1.06	1.00	0.96–1.05

Model 1: adjusted for age and sex

Model 2: Model 1 + smoking status, education, total energy (kcal/day), vitamin supplementation, body mass index (kg/m^2^), waist circumference (cm), cycling, sports, prevalent hypertension (not in the analyses on cancer), alcohol intake (7 categories) (only for the Nordic diet analysis)

*SD* standard deviation, *HR* hazard ratio, *CI* confidence interval

*tMDS* Mediterranean diet score based on that established by Trichopoulou et al. [30], *MedPyr* Mediterranean diet score based on the Mediterranean Pyramid

**Table 6:** Prospective associations between the diet scores and certain disease outcomes stratified by sex in the EPIC-Potsdam cohort

	Low adherence	Moderate adherence		High adherence		*p* for trend	per SD		per 1 unit		*p* for interaction
	(Ref.)	HR	95% CI	HR	95% CI		HR	95% CI	HR	95% CI	
Nordic diet score and stroke											0.071
Men											
Cases, n/person-year	63/31,471	60/33,485		52/32,401							
Fully adjusted model	1.00	0.95	0.65–1.37	0.85	0.56–1.29	0.406	0.88	0.74–1.04	0.96	0.91–1.01	
Women											
Cases, n/person-year	45/50,458	40/54,556		61/50,359							
Fully adjusted model	1.00	0.72	0.46–1.12	1.05	0.68–1.64	0.913	1.06	0.87–1.28	1.02	0.96–1.08	
Mediterranean pyramid score and myocardial infarction											0.070
Men											
Cases, n/person-year	82/31,889	75/32,219		61/32,325							
Fully adjusted model	1.00	0.97	0.71–1.34	0.87	0.62–1.22	0.397	0.98	0.86–1.13	0.99	0.88–1.10	
Women											
Cases, n/person-year	41/51,641	29/52,014		24/52,174							
Fully adjusted model	1.00	0.80	0.50–1.30	0.77	0.45–1.30	0.313	0.81	0.65–1.00	0.84	0.71–1.00	

Fully adjusted model: age, sex, smoking status, education, total energy (kcal/day), vitamin supplementation, body mass index (kg/m^2^), waist circumference (cm), cycling, sports, prevalent hypertension, alcohol intake (7 categories) (only for the Nordic diet analysis)

*SD* standard deviation, *HR* Hazard ratio, *CI* confidence interval

**Table 7:** Prospective associations of the diet scores with type 2 diabetes mellitus and myocardial infarction, excluding single components

Diabetes						Myocardial infarction in women	
	tMDS		MedPyr			MedPyr	
	HR (per 1 SD)	95% CI		HR (per 1 SD)	95% CI	HR (per 1SD)	95% CI
Overall association	0.93	0.88–0.98	Overall association	0.92	0.87–0.97	0.81	0.65–1.00
Minus cereals	0.93	0.88–0.98	Minus cereals	0.91	0.87–0.96	0.81	0.66–1.00
Minus fruits and nuts	0.94	0.89–0.99	Minus fruits	0.92	0.87–0.97	0.81	0.66–1.00
			Minus nuts	0.92	0.87–0.97	0.82	0.67–1.02
Minus vegetables	0.94	0.89–1.00	Minus vegetables	0.92	0.87–0.97	0.81	0.65–1.00
Minus fish	0.92	0.87–0.98	Minus fish	0.91	0.86–0.96	0.82	0.67–1.02
Minus legumes	0.92	0.87–0.98	Minus legumes	0.91	0.86–0.96	0.80	0.65–0.99
Minus meat	0.94	0.89–1.00	Minus red meat	0.93	0.88–0.98	0.86	0.70–1.07
			Minus processed meat	0.92	0.88–0.98	0.80	0.65–0.99
			Minus white meat	0.91	0.87–0.97	0.81	0.65–1.00
			Minus egg	0.91	0.86–0.96	0.76	0.62–0.94
Minus dairy products	0.92	0.87–0.98	Minus dairy products	0.93	0.88–0.98	0.79	0.64–0.98
			Minus potatoes	0.93	0.88–0.98	0.86	0.69–1.06
Minus alcohol	0.95	0.90–1.01	Minus alcohol	0.94	0.89–0.99	0.88	0.71–1.08
			Minus sweets	0.91	0.86–0.96	0.83	0.67–1.02
Minus olive oil	0.95	0.89–1.00	Minus olive oil	0.91	0.87–0.97	0.77	0.62–0.94

Age, sex, smoking status, education, total energy (kcal/day), vitamin supplementation, body mass index (kg/m^2^), waist circumference (cm), cycling, sports, prevalent hypertension, excluded component

*CI* confidence interval, *HR* hazard ratio, *SD* standard deviation

*tMDS* Mediterranean diet score based on that established by Trichopoulou et al. [30]]

*MedPyr* Mediterranean diet score based on the Mediterranean Pyramid

## Discussion

In this study, we have analyzed the association between the MedDiet and the Nordic diet with the incidence of major chronic disease in a German, middle aged, and apparently healthy population. With a mean of 10.6 years of follow-up, the two different MedDiet scores showed inverse associations with the risk of developing T2D. We also observed an inverse association of the MedPyr score and the risk of MI among women. The Nordic diet showed an inverse but not-statistically significant association with MI risk in the overall population, and with stroke in men. No association was observed for any of the scores with the incidence of cancer.

To our knowledge, our analyses are the first to explore the associations between the Nordic diet and several different major chronic diseases in a prospective cohort outside of the Nordic region. Three large prospective cohorts have studied the effects of the Nordic diet on the onset of major chronic diseases. In the Diet, Cancer and Health study in Denmark, which included more than 55,000 men and women and followed them for more than 13 years, a higher adherence to the Nordic diet was associated with lower risk for total stroke, T2D, and colorectal cancer, the latter only among women [3, 5, 6]. The Swedish Women’s Lifestyle and Health cohort included more than 44,000 women followed for over a median of 20 years [10]; however, no association between the Nordic diet and the risk of breast and colorectal cancer or CVD was observed [7, 9, 33]. In addition, in two Finnish prospective cohorts, the Helsinki Birth Cohort and the Health 2000 survey, no association between the Nordic diet and the risk of T2D was detected [4].

The construction of the Nordic diet score is probably the most important difference between previous studies and our present study. The Diet, Cancer and Health study [5] and the Swedish Women’s Lifestyle and Health cohort [10] used a very similar index composed of six food groups, namely oatmeal, apples/pears, cabbages, root vegetables, fish, and rye bread in the Danish Diet, Cancer and Health cohort [5], but whole grain cereals in the Swedish Women’s Lifestyles and Health study [10]. However, it would be too ambitious to hypothesize that this small disparity could lead to the different observations. The Helsinki Birth Cohort and the Health 2000 survey indicated no association between the Nordic diet and the risk of T2D, considering six components in line with the Nordic diet pyramid [4] (berries/apples/pears, vegetables, rye/oats/barley, fat-free milk/< 2% fat milk, salmon/freshwater fish, and polyunsaturated fatty acids/(saturated fatty acids + *trans*-fatty acids) ratio), two components that contradict these (meat products and total fat (E%)), and alcohol, which should be consumed in moderation. Again, the composition of the pattern does not provide a possible explanation for this null association. Indeed, previous publications in the same cohorts already showed an inverse association of the Nordic diet with abdominal obesity [34] and low-grade inflammation [35]. Other items considered as part of the Nordic diet in other publications, like potatoes, low-fat dairy products and vegetable oils, were also included in our score [26, 36]. These discrepancies highlight the necessity to get into an agreement about the definition of Nordic diet.

Regarding the MedDiet, beneficial effects on the incidence of T2D have already been reported in a large randomized control trial, the PREDIMED study [15], as well as in large longitudinal cohorts, and summarized in several meta-analyses [17–19]. Moreover, we have shown that alcohol presented the highest contribution to the observed beneficial effect of both MedDiet scores. This observation is in line with EPIC-InterAct study, which also observed that high olive oil and low meat consumption, besides moderate alcohol consumption, are important components of the effect of the MedDiet on T2D. In line with these observations, a meta-analysis concluded that olive oil could have beneficial effects on the prevention and management of T2D [37]. Finally, another meta-analysis also determined that the intake of red and processed meat increased the risk. In our study, we also showed a marginal effect of these two components [38]. The association between MedDiet and the risk of CVD has been extensively explored [12–14, 39]. In our analysis, we observed a tendency of adherence to the MedPyr to lower the risk for incident MI, with a stronger effect in women than in men. Similarly to T2D, this association appeared to be at least in part attributable to alcohol intake. The protective effects of alcohol intake on cardiovascular risk have been a matter of substantial debate. A recent meta-analysis supported the idea that the health effects of alcohol intake depend on the amount as well as on the pattern of consumption [40]. Nevertheless, a recent study from Bell et al. [41] found heterogeneous associations for the level of alcohol consumption and different cardiovascular endpoints, encouraging for a more accurate and careful research and public health counseling.

In our study, we observed sex differences in the association of the a priori defined scores with CVD. Previous studies have already discussed the sex differences in response to diet, particularly fat-rich diets [42–44]. Grosso et al. [13] summarized prospective studies in a meta-analysis and observed an inverse association between adherence to the MedDiet and the occurrence of stroke. Recently, a work evaluated the associations of four different MedDiet scores with the risk of CVD in the EPIC-Norfolk prospective cohort [14]. An inverse association was observed for stroke with the MedPyr score but not for the three other scores assessed. The construction of the dietary scores (items included and cut-offs used) may directly affect the results. This is one of the major critic points and has been extensively discussed before [21, 45–47]. In this study, we decided to use one of the most frequently applied scores to represent the MedDiet in non-Mediterranean areas, the tMDS. For the calculation of this score, we used population-specific cut-off values for most component food groups, thus assigning score points based on relative ranking in the cohort rather than absolute food consumption levels. Another study conducted in the EPIC-Potsdam cohort evaluating the effects of the MedDiet on heart failure [48] suggested the use of observed medians in the EPIC-Greece cohort [30], arguing that, by using these, the calculated score better represents food consumption levels of the ‘true’ MedDiet. Apart from this, and following the suggestion from Tong et al. [14], we also used the MedPyr score, which is based on the Mediterranean pyramid proposed by the Mediterranean Diet Foundation [11]. This score has two major differences in comparison to the tMDS. First, the pyramid includes more food groups (15) – separating fruits and nuts, distinguishing between red, processed and white meat, and additionally including potatoes, eggs, and sweets. Second, score points are assigned to absolute food consumption levels rather than relative intake. For those food items included in both scores, the MedPyr score reflected somewhat smaller differences in absolute intake between those considered to have a high versus low score compared to tMDS (Additional file 1: Table S1). Both differences in the definition of components and in their intake levels are possible explanations for inconsistent associations observed for both scores in our cohort. The study of Tong et al. [14], in line with our observations, also showed the strongest associations with cardiovascular outcomes for the MedPyr score. However, they also conclude that the three scores used to assess adherence to the MedDiet (the MedPyr, the tMDs, and a third one based on the literature as proposed by Sofi et al. [49]) were useful in epidemiological settings [14]. Unfortunately, thus far, there are no data available regarding the utility and the use of different Nordic diet scores. On the other hand, for a better comparability among the created scores and with other previous publications, we decided to construct scores without adjusting components for total energy intake. We included total energy intake as a covariate in the regression models in order to achieve the equivalent of an isocaloric diet. This is in accordance with the methodology applied by Trichopoulou et al. [30, 50] when they first created and evaluated the MedDiet score and its implication on health. Still, the role of energy adjustment of components prior to dietary pattern score calculation remains a matter of scientific debate. First, heterogeneity exists across previous studies as to whether and what kind of energy adjustments were applied, which limits the comparability between studies. Secondly, few studies have been performed to evaluate the consequences of such methodological differences. For example, studies have found little difference in derived patterns if input variables were or not adjusted for energy. Additionally, expressing the input variables as contributions to energy intake might be less meaningful for food groups that might be important for health but contribute little to energy intake [51].

Some strengths and limitations of the present work should be mentioned. The EPIC-Potsdam study is a large follow-up study that includes a high number of apparently healthy participants at baseline. Together with the medical verification of the new incident cases, the validity of our observations was strengthened. On the other hand, the use of a FFQ as a tool for measuring habitual dietary intake is not exempt from limitations and there is much discussion on how well the FFQ captures habitual diet [52, 53]. For example, misreporting is a common problem, which could lead to measurement error and bias in the estimations. Moreover, diet and other lifestyle factors were only assessed at baseline, and possible changes have not been taken into account. Possible diet changes after the diagnosis of a chronic disease could have led to reverse causality and thus explain our observations. However, after the exclusion of prevalent cases of chronic disease and the further sensitivity analysis excluding incident cases within the first 2 years, we made an attempt to minimize this problem. We have also controlled our analysis for a large number of plausible confounders that could influence the association between the dietary patterns and risk of chronic diseases. Nevertheless, residual confounding is possible due to imperfect measurement or presence of unknown confounders. Due to possible limited power, we decided to combine overall cancer incidence as an outcome. However, this approach might complicate possible conclusions and interpretations of the null finding and, still, possible beneficial effects on specific cancer sites cannot be ruled out. The same problem on limited power could also apply to incident stroke and MI.

## Conclusions

In summary, we have observed an inverse association of both MedDiet scores with the incidence of T2D, with alcohol being a relevant component within these. At the same time, we have observed that the MedPyr score was also associated with lower risk of MI in women, but not in men. However, we have not observed any other statistically significant association between the evaluated scores and the risk of stroke or overall cancer. Nevertheless, possible beneficial effects cannot be completely ruled out due to limited power to evaluate associations and the possibility of cancer site-specific effects. Thus, re-analysis of the associations in our cohort after extended follow-up would include a higher number of incident cases and provide further information.

## Additional file

Additional file 1: **Table S1.** Diet components of the EPIC-Potsdam population according to degree of adherence to the diet scores. **Table S2.** Prospective associations between adherence to the three diet scores and the incidence of major chronic diseases (T2D, MI, stroke, and overall cancer) in the EPIC-Potsdam cohort. Excluding cases in the first 2 years. **Table S3.** Prospective associations between adherence to the three diet scores and the incidence of stroke, excluding hemorrhagic stroke cases, in the EPIC-Potsdam cohort. (DOCX 47 kb)

## Abbreviations

CI: confidence interval
CVD: cardiovascular disease
EPIC: European Prospective study into Cancer and Nutrition
FFQ: food frequency questionnaire
HR: hazard ratio
MedPyr: Mediterranean pyramid score
MI: myocardial infarction
PREDIMED: Prevención con dieta mediterránea
T2D: type 2 diabetes
tMED: traditional Mediterranean diet score
