# Supplementary material for: Nordic diet, Mediterranean diet, and the risk of chronic diseases: the EPIC-Potsdam study
Source: BMC Med. 2018 Jun 27;16:99. doi: 10.1186/s12916-018-1082-y (PMC6020433; doi:10.1186/s12916-018-1082-y)
Supplement: Supplementary file 1 — Table S1. Diet components of the EPIC-Potsdam population according to degree of adherence to the diet scores. Table S2. Prospective associations between adherence to the three diet scores and the incidence of major chronic diseases (T2D, MI, stroke, and overall cancer) in the EPIC-Potsdam cohort. Excluding cases in the first 2 years. Table S3. Prospective associations between adherence to the three diet scores and the incidence of stroke, excluding hemorrhagic stroke cases, in the EPIC-Potsdam cohort. (DOCX 47 kb) [file 12916_2018_1082_MOESM1_ESM.docx]

**Additional file 1**

**Table S1**: Diet components of the EPIC-Potsdam population according to degree of adherence to the diet scores

|  | Total population  (n=23.485) | Nordic diet score | | tMDS | | MedPyr score | |
| --- | --- | --- | --- | --- | --- | --- | --- |
|  |  | Low adherence (n=7.686)  0-7 points | High adherence (n=7.637)  11-18 points | Low adherence (n=7.077)  0-7 points | High adherence (n=6.709)  11-18 points | Low adherence (n=7.828)  < 6.2 points | High adherence (n=7.828)  > 7.3 points |
| Nordic Diet score | 9.0 (3.1) | 5.5 (1.5) | 12.5 (1.5) | 7.4 (2.9) | 10.6 (2.9) | 8.2 (3.1) | 9.9 (3.0) |
| tMDS score | 9.0 (2.7) | 7.7 (2.4) | 10.2 (2.5) | 5.8 (1.3) | 12.2 (1.3) | 7.1 (2.2) | 10.9 (2.2) |
| MedPyr score | 6.8 (1.2) | 6.4 (1.2) | 7.1 (1.2) | 5.8 (1.0) | 7.7 (1.0) | 5.4 (0.7) | 8.1 (0.6) |
| Total energy intake (kcal/day) | 2127 (671) | 1839 (574) | 2433 (683) | 2011 (663) | 2250 (664) | 2040 (687) | 2222 (660) |
| Fibre (g/1000 kcal) | 10.8 (2.7) | 10.1 (2.7) | 11.5 (2.6) | 9.8 (2.4) | 11.8 (2.7) | 10.5 (2.7) | 11.2 (2.7) |
| Protein (energy %) | 14.6 (2.2) | 14.3 (2.3) | 14.9 (2.1) | 14.7 (2.4) | 14.5 (2.1) | 14.6 (2.3) | 14.7 (2.1) |
| Protein from plants (g/day) | 26.5 (8.3) | 22.9 (7.3) | 30.3 (8.2) | 23.8 (7.9) | 29.3 (8.1) | 25.3 (8.5) | 27.9 (8.1) |
| CHO (energy %) | 45.4 (6.3) | 45.0 (6.9) | 45.7 (5.8) | 45.1 (6.8) | 45.7 (5.8) | 46.0 (6.8) | 44.8 (5.8) |
| Fat (energy %) | 34.5 (5.4) | 34.5 (5.9) | 34.6 (4.9) | 35.3 (5.8) | 33.9 (5.0) | 34.6 (5.8) | 34.3 (5.0) |
| PUFA (energy %) | 6.2 (1.8) | 5.9 (1.8) | 6.6 (1.6) | 6.1 (1.9) | 6.3 (1.6) | 6.2 (1.9) | 6.3 (1.6) |
| SFA (energy %) | 14.4 (2.9) | 14.6 (3.2) | 14.1 (2.6) | 15.0 (3.1) | 13.8 (2.7) | 14.6 (3.1) | 14.1 (2.7) |
| MUFA (energy %) | 12.1 (2.1) | 12.1 (2.2) | 12.1 (1.9) | 12.3 (2.2) | 12.0 (2.0) | 12.0 (2.2) | 12.2 (2.0) |
| Cereals (g/day)* | 190.4 (143.5-242.1) | 161.9 (119.3-211.5) | 217.7 (173.7-271.0) | 164.3 (122.0-212.2) | 214.9 (168.5-266.9) | 175.4 (126.1-229.9) | 203.3 (160.1-251.6) |
| Whole grain cereals (g/day)* | 133.2 (89.1-184.7) | 100.2 (68.8-148.6) | 163.8 (118.2-210.2) | 106.8 (74.3-158.1) | 156.0 (106.8-204.7) | 117.4 (77.4-176.4) | 144.2 (100.2-191.2) |
| Vegetables (g/day)* | 90.9 (65.1-123.4) | 69.5 (48.9-93.6) | 114.1 (87.2-152.7) | 68.7 (50.2-91.0) | 119.0 (91.8-154.1) | 82.2 (57.6-112.6) | 99.8 (73.8-138.6) |
| Cabbage/cruciferous (g/day)* | 16.2 (9.7-26.0) | 10.2 (6.3-16.2) | 24.1 (15.9-34.7) | 12.9 (7.5-21.2) | 20.0 (12.4-30.9) | 15.8 (9.0-26.1) | 16.8 (10.3-26.4) |
| Root vegetables (g/day)* | 9.1 (5.1-15.6) | 5.4 (3.1-8.7) | 14.8 (9.4-22.5) | 6.9 (3.8-11.7) | 11.9 (7.2-20.2) | 7.9 (4.4-13.6) | 10.6 (6.1-18.1) |
| Fruits (g/day)* | 100.5 (81.8-188.8) | 89.3 (55.7-132.4) | 172.5 (95.6-231.4) | 89.0 (60.0-121.5) | 177.2 (97.4-227.5) | 94.4 (68.8- 154.9) | 149.6 (91.6-214.1) |
| Berries (g/day)* | 0.9 (0.2-3.1) | 0.3 (0.0-1.3) | 2.4 (0.7-5.4) | 0.5 (0.1-2.0) | 1.6 (0.4-4.5) | 0.6 (0.1-2.4) | 1.4 (0.3-3.9) |
| Apple and pear (g/day)* | 28.3 (13.4-54.0) | 15.3 (7.1-30.9) | 46.3 (26.2-74.7) | 18.9 (8.3-36.5) | 41.7 (22.5-71.0) | 22.9 (10.2-44.5) | 35.3 (18.2-65.6) |
| Nuts (g/day)* | 0.8 (0.4-4.1) | 0.8 (0.4-3.6) | 0.8 (0.4-4.1) | 0.8 (0.4-2.1) | 0.8 (0.4-4.1) | 0.4 (0.4-2.1) | 0.8 (0.4-4.1) |
| Total dairy products (g/day)* | 184.3 (106.1-300.0) | 127.9 (78.0-208.2) | 252.3 (160.4-380.3) | 212.2 (121.3-348.8) | 160.4 (97.6-255.8) | 152.9 (81.3-286.3) | 207.2 (134.3-311.5) |
| Low fat dairy prod. (g/day)* | 175.3 (96.3-291.4) | 117.4 (67.6-198.5) | 245.2 (152.9-371.7) | 203.9 (112.6-341.1) | 151.2 (88.4-247.8) | 143.2 (71.8-277.3) | 198.7 (124.4-303.2) |
| Potatoes (g/day)* | 88.8 (55.6-127.5) | 66.1 (42.0-100.7) | 110.4 (77.5-145.0) | 81.8 (49.5-124.6) | 93.5 (61.9-129.1) | 111.1 (67.8-146.8) | 74.2 (50.1-104.4) |
| Meat (g/day)* | 101.8 (70.8-145.5) | 90.2 (62.4-129.2) | 115.0 (80.9-162.1) | 106.3 (73.8-146.4) | 97.0 (65.3-140.8) | 103.5 (71.8-151.1) | 100.8 (69.5-141.3) |
| White meat (g/day)* | 9.0 (4.4-17.1) | 7.0 (3.6-13.5) | 11.5 (5.9-20.3) | 7.9 (4.0-15.5) | 9.9 (4.9-18.2) | 7.1 (3.8-13.4) | 11.8 (5.8-20.5) |
| Red meat (g/day)* | 35.6 (22.6-53.8) | 31.5 (18.6-46.6) | 40.3 (26.1-60.6) | 34.8 (22.5-53.1) | 35.3 (21.6-52.9) | 39.2 (24.0-63.8) | 33.2 (21.4-46.7) |
| Processed meat (g/day)* | 49.3 (31.4-78.1) | 45.4 (27.3-69.1) | 53.8 (36.1-86.4) | 51.8 (34.7-82.7) | 46.4 (27.5-69.9) | 49.0 (33.1-76.2) | 49.0 (29.3-79.4) |
| Fish (g/day)* | 18.4 (9.9-29.0) | 11.2 (4.6-21.4) | 27.3 (16.4-39.9) | 11.2 (4.6-21.4) | 27.3 (16.4-39.9) | 12.3 (4.6-22.5) | 25.6 (16.4-39.9) |
| Egg (g/day)* | 14.5 (9.0-21.9) | 13.6 (7.8-21.8) | 16.0 (9.0-25.3) | 13.6 (9.0-21.9) | 16.0 (9.0-24.5) | 10.3 (5.7-20.2) | 17.8 (10.3-25.3) |
| Legumes (g/day)* | 17.0 (9.1-32.0) | 12.8 (7.0-25.4) | 22.4 (12.1-36.6) | 11.3 (6.7-20.9) | 25.9 (14.1-39.3) | 14.2 (8.2-26.6) | 22.0 (10.8-36.4) |
| Sweets (g/day)* | 80.7 (46.4-131.6) | 67.9 (37.3-113.0) | 95.1 (57.2-149.7) | 77.5 (44.3-129.1) | 84.9 (49.7-134.4) | 79.0 (46.7-129.5) | 82.4 (45.0-133.0) |
| Vegetable fats^‡^ (g/day)* | 15.4 (7.0-25.4) | 10.6 (3.9-21.1) | 22.7 (12.6-33.8) | 13.7 (5.4-23.8) | 16.7 (8.5-27.4) | 14.9 (6.2-24.6) | 16.3 (8.0-26.2) |
| Olive oil (g/day)* | 0.3 (0.0-1.5) | 0.2 (0.0-1.2) | 0.5 (0.0-1.9) | 0.0 (0.0-0.4) | 1.4 (0.3-3.0) | 0.0 (0.0-0.3) | 1.0 (0.3-2.5) |
| Alcohol (g/day)* | 8.3 (3.1-19.3) | 8.2 (2.8-20.1) | 8.6 (3.4-18.8) | 4.2 (1.6-11.6) | 11.8 (6.5-21.6) | 4.0 (1.5-11.4) | 12.1 (7.0-21.5) |

Data are shown as mean (SD) unless otherwise stated. *Data are shown as median (IQR). IQR: Interquartile range. ^‡^Excluding olive oil. PUFA: Polyunsaturated fatty acids. SFA: Saturated fatty acids. MUFA: Monounsaturated fatty acids. tMDS: Mediterranean diet score based on the one created by Trichopoulou et al. MedPyr: Mediterranean diet score based on the Mediterranean Pyramid

**Table S2**: Prospective associations between adherence to the three diet scores and the incidence of major chronic diseases (T2D, MI, stroke and overall cancer) in the EPIC-Potsdam cohort. Excluding cases in the first 2 years

|  | Low adherence | Moderate adherence | | High  adherence | | *p* for trend | per 1 SD | | per 1 unit | |
| --- | --- | --- | --- | --- | --- | --- | --- | --- | --- | --- |
|  | (Ref.) | HR | 95% CI | HR | 95% CI |  | HR | 95% CI | HR | 95% CI |
| DIABETES | | | | | | | | | | |
| Nordic diet | | | | | | | | | | |
| Cases, n/person-year | 343/79932 | 417/85359 | | 392/80521 | |  |  |  |  |  |
| Model 1 | 1.00 | 1.05 | 0.91-1.22 | 1.00 | 0.87-1.16 | 0.851 | 0.99 | 0.93-1.05 | 1.00 | 0.98-1.02 |
| Model 2 | 1.00 | 1.04 | 0.90-1.21 | 1.04 | 0.88-1.22 | 0.976 | 1.00 | 0.93-1.07 | 1.00 | 0.98-1.02 |
| tMDS | | | | | | | | | | |
| Cases, n/person-year | 380/73802 | 477/101530 | | 295/70481 | |  |  |  |  |  |
| Model 1 | 1.00 | 0.84 | 0.74-0.96 | 0.74 | 0.63-0.86 | <0.001 | 0.87 | 0.82-0.93 | 0.95 | 0.93-0.97 |
| Model 2 | 1.00 | 0.90 | 0.78-1.03 | 0.83 | 0.71-0.97 | 0.012 | 0.92 | 0.87-0.98 | 0.97 | 0.95-0.99 |
| MedPyr | | | | | | | | | | |
| Cases, n/person-year | 470/81006 | 366/82024 | | 316/82783 | |  |  |  |  |  |
| Model 1 | 1.00 | 0.80 | 0.70-0.92 | 0.71 | 0.62-0.82 | <0.001 | 0.87 | 0.82-0.92 | 0.89 | 0.85-0.93 |
| Model 2 | 1.00 | 0.85 | 0.74-0.98 | 0.78 | 0.68-0.91 | 0.001 | 0.90 | 0.85-0.96 | 0.92 | 0.88-0.97 |
| MYOCARDIAL INFARCTION | | | | | | | | | | |
| Nordic diet |  |  |  |  |  |  |  |  |  |  |
| Cases, n/person-year | 79/81906 | 106/87486 | | 81/82668 | |  |  |  |  |  |
| Model 1 | 1.00 | 1.13 | 0.85-1.52 | 0.86 | 0.63-1.17 | 0.269 | 0.90 | 0.80-1.02 | 0.97 | 0.93-1.01 |
| Model 2 | 1.00 | 1.13 | 0.84-1.53 | 0.85 | 0.60-1.20 | 0.280 | 0.89 | 0.77-1.02 | 0.96 | 0.92-1.00 |
| tMDS |  |  |  |  |  |  |  |  |  |  |
| Cases, n/person-year | 78/75835 | 120/104155 | | 68/72070 | |  |  |  |  |  |
| Model 1 | 1.00 | 1.01 | 0.76-1.35 | 0.80 | 0.57-1.10 | 0.193 | 0.89 | 0.79-1.01 | 0.96 | 0.92-1.00 |
| Model 2 | 1.00 | 1.09 | 0.81-1.45 | 0.89 | 0.64-1.24 | 0.521 | 0.93 | 0.82-1.06 | 0.97 | 0.93-1.02 |
| MedPyr |  |  |  |  |  |  |  |  |  |  |
| Cases, n/person-year | 100/83417 | 95/84077 | | 71/84566 | |  |  |  |  |  |
| Model 1 | 1.00 | 0.98 | 0.74-1.30 | 0.80 | 0.56-1.08 | 0.192 | 0.88 | 0.78-0.99 | 0.90 | 0.82-1.00 |
| Model 2 | 1.00 | 1.01 | 0.76-1.34 | 0.85 | 0.62-1.16 | 0.399 | 0.91 | 0.80-1.03 | 0.93 | 0.84-1.02 |
| STROKE | | | | | | | | | | |
| Nordic diet |  |  |  |  |  |  |  |  |  |  |
| Cases, n/person-year | 96/81872 | 89/87662 | | 105/82724 | |  |  |  |  |  |
| Model 1 | 1.00 | 0.77 | 0.58-1.03 | 0.90 | 0.68-1.19 | 0.429 | 0.93 | 0.83-1.05 | 0.98 | 0.94-1.02 |
| Model 2 | 1.00 | 0.82 | 0.61-1.11 | 1.02 | 0.74-1.40 | 0.952 | 0.98 | 0.86-1.12 | 0.99 | 0.95-1.04 |
| tMDS |  |  |  |  |  |  |  |  |  |  |
| Cases, n/person-year | 84/75765 | 128/104337 | | 78/72155 | |  |  |  |  |  |
| Model 1 | 1.00 | 1.02 | 0.78-1.35 | 0.89 | 0.65-1.21 | 0.625 | 0.98 | 0.88-1.11 | 0.99 | 0.95-1.04 |
| Model 2 | 1.00 | 1.06 | 0.80-1.39 | 0.95 | 0.69-1.31 | 0.987 | 1.01 | 0.90-1.14 | 1.00 | 0.96-1.05 |
| MedPyr |  |  |  |  |  |  |  |  |  |  |
| Cases, n/person-year | 115/83525 | 81/84214 | | 94/84519 | |  |  |  |  |  |
| Model 1 | 1.00 | 0.75 | 0.56-0.99 | 0.94 | 0.71-1.23 | 0.632 | 0.98 | 0.87-1.10 | 0.98 | 0.89-1.08 |
| Model 2 | 1.00 | 0.78 | 0.58-1.03 | 1.00 | 0.75-1.32 | 0.989 | 1.01 | 0.89-1.13 | 1.01 | 0.91-1.11 |
| CANCER | | | | | | | | | | |
| Nordic diet |  |  |  |  |  |  |  |  |  |  |
| Cases, n/person-year | 460/79333 | 512/84913 | | 518/79800 | |  |  |  |  |  |
| Model 1 | 1.00 | 0.93 | 0.82-1.06 | 0.95 | 0.84-1.08 | 0.296 | 0.97 | 0.92-1.02 | 0.99 | 0.97-1.01 |
| Model 2 | 1.00 | 0.95 | 0.83-1.08 | 0.99 | 0.86-1.14 | 0.592 | 0.98 | 0.93-1.04 | 0.99 | 0.98-1.01 |
| tMDS |  |  |  |  |  |  |  |  |  |  |
| Cases, n/person-year | 449/73547 | 622/100634 | | 419/69864 | |  |  |  |  |  |
| Model 1 | 1.00 | 0.95 | 0.84-1.07 | 0.92 | 0.80-1.05 | 0.165 | 0.97 | 0.92-1.03 | 0.99 | 0.97-1.01 |
| Model 2 | 1.00 | 0.96 | 0.85-1.09 | 0.96 | 0.85-1.09 | 0.350 | 0.99 | 0.94-1.04 | 1.00 | 0.98-1.02 |
| MedPyr diet |  |  |  |  |  |  |  |  |  |  |
| Cases, n/person-year | 524/80638 | 509/81468 | | 457/81939 | |  |  |  |  |  |
| Model 1 | 1.00 | 1.03 | 0.91-1.16 | 0.97 | 0.86-1.10 | 0.912 | 0.99 | 0.94-1.04 | 0.99 | 0.95-1.04 |
| Model 2 | 1.00 | 1.04 | 0.92-1.17 | 1.00 | 0.88-1.14 | 0.788 | 1.01 | 0.95-1.06 | 1.01 | 0.96-1.05 |

Model 1: adjusted for age and sex

Model 2: Model 1 + smoking status, education, total energy (kcal/day), vitamin suppl., BMI (kg/m²), waist circumference (cm), cycling, sports, prevalent HT, alcohol intake (7 categories) (only for the Nordic diet analysis)

SD: standard deviation; HR: Hazard ratios; CI: Confidence interval

tMDS: Mediterranean diet score based on the one created by Trichopoulou et al. MedPyr: Mediterranean diet score based on the Mediterranean Pyramid

**Table S3**: Prospective associations between adherence to the three diet scores and the incidence stroke, excluding haemorrhagic stroke cases, in the EPIC-Potsdam cohort.

Model 1: adjusted for age and sex

|  | Low adherence | Moderate adherence | | High  adherence | | *p* for trend | per 1 SD | | per 1 unit | |
| --- | --- | --- | --- | --- | --- | --- | --- | --- | --- | --- |
|  | (Ref.) | HR | 95% CI | HR | 95% CI |  | HR | 95% CI | HR | 95% CI |
| STROKE | | | | | | | | | | |
| Nordic diet | | | | | | | | | | |
| Cases, n/person-year | 93/81806 | 81/87539 | | 89/82521 | |  |  |  |  |  |
| Model 1 | 1.00 | 0.72 | 0.54-0.98 | 0.78 | 0.58-1.04 | 0.099 | 0.89 | 0.78-1.00 | 0.96 | 0.93-1.00 |
| Model 2 | 1.00 | 0.79 | 0.58-1.07 | 0.93 | 0.67-1.30 | 0.638 | 0.96 | 0.84-1.11 | 0.99 | 0.94-1.03 |
| tMDS | | | | | | | | | | |
| Cases, n/person-year | 74/75618 | 121/10421 | | 68/72038 | |  |  |  |  |  |
| Model 1 | 1.00 | 1.09 | 0.81-1.45 | 0.88 | 0.63-1.22 | 0.436 | 0.96 | 0.85-1.09 | 0.99 | 0.94-1.03 |
| Model 2 | 1.00 | 1.15 | 0.86-1.54 | 0.98 | 0.70-1.38 | 0.984 | 1.01 | 0.89-1.15 | 1.00 | 0.96-1.05 |
| MedPyr | | | | | | | | | | |
| Cases, n/person-year | 105/83423 | 76/84085 | | 82/84358 | |  |  |  |  |  |
| Model 1 | 1.00 | 0.77 | 0.57-1.03 | 0.90 | 0.68-1.21 | 0.441 | 0.93 | 0.83-1.05 | 0.95 | 0.86-1.04 |
| Model 2 | 1.00 | 0.82 | 0.61-1.11 | 1.01 | 0.75-1.37 | 0.982 | 0.98 | 0.87-1.12 | 0.99 | 0.89-1.09 |

Model 2: Model 1 + smoking status, education, total energy (kcal/day), vitamin suppl., BMI (kg/m²), waist circumference (cm), cycling, sports, prevalent hypertension, alcohol intake (7 categories) (only for the Nordic diet analysis)

SD: standard deviation; HR: Hazard ratios; CI: Confidence intervals

tMDS: Mediterranean diet score based on the one created by Trichopoulou et al. MedPyr: Mediterranean diet score based on the Mediterranean Pyramid
